# Climate change and eye health: Awareness of health sciences students at a South African University

**DOI:** 10.4102/hsag.v31i0.3115

**Published:** 2026-01-13

**Authors:** Hlabje C. Masemola, Lara Bakker, Chizelle Spies, Waitse Mmono, Carlynne Christians, Chanelle Meintjes, Omololu Aluko

**Affiliations:** 1 Department of Optometry, Faculty of Health Sciences, University of the Free State, Bloemfontein, South Africa; 2 Department of Biostatistics, Faculty of Health Sciences, University of the Free State, Bloemfontein, South Africa

**Keywords:** climate change, ocular health, knowledge, perception, public health

## Abstract

**Background:**

Climate change is an increasingly global issue with significant adverse impacts on public health. Its effects can lead to an increase in ocular health problems and diseases.

**Aim:**

To determine the knowledge and perception among final-year students registered in the School of Health and Rehabilitation Sciences at the University of the Free State Bloemfontein, on the impact of climate change on eye health.

**Setting:**

The study was conducted at the University of the Free State, Bloemfontein campus.

**Methods:**

A descriptive study was carried out using a self-administered questionnaire. Responses were collected during June 2024 and July 2024, and the results were analysed using descriptive statistics using SAS version 15.2.

**Results:**

A total of 107 final-year health science students participated in the study, with a response rate of 85.60%. The study found a high level of awareness about climate change, with 99.07% (*n* = 106) acknowledging its occurrence. However, 64.49% (*n* = 69) of the participants felt they were not well informed about climate change issues, while 35.51% (*n* = 38) felt adequately informed. In addition, 93.46% of participants believed that protecting their eyes outdoors can reduce the risk of climate change-related eye health issues.

**Conclusion:**

The study highlights the varying levels of knowledge among future healthcare professionals, emphasising the need for enhanced educational initiatives to bridge gaps in understanding.

**Contribution:**

This study adds to the broader public health conversation by highlighting the connection between specific environmental changes and eye health. It calls for detailed approaches to reduce the negative impacts of climate change on eye health.

## Introduction

Climate change is an increasingly global issue with significant negative impacts on public health (United Nation 2023a). Climate change is a long-term change to weather and temperature patterns, and such changes may occur naturally as a result of variations in the sun’s activity or significant volcanic eruptions (Intergovernmental Panel on Climate Change [IPCC] 2007; United Nation 2023a). However, in recent times, these changes have been largely driven by human activities, particularly the burning of fossil fuels such as coal, oil and gas. It has been reported that climate change exacerbates the environmental and social determinants of health, such as food insecurity and water scarcity, leading to malnutrition and dehydration (Ahmed 2011). These conditions can indirectly affect eye health by increasing the risk of vitamin A deficiency-related conditions such as xerophthalmia and night blindness compromising the immune system, making the eyes more susceptible to infections (Ahmed 2011). It also has a significant impact on health and generates negative impacts on human health affecting every system in the body (World Health Organization [WHO] 2023). While much attention has been given to its impacts on physical health and the environment, its effects on eye health remain relatively understudied and poorly understood (WHO 2023). According to the International Agency for the Prevention of Blindness, the devastating impact of climate change will affect the eye leading to an increase in the prevalence of eye injuries, age-related macular degeneration, trachoma infections, cataracts, eye lesions, severe allergic eye illnesses and glaucoma (International Agency for the Prevention of Blindness 2024).

Climate change significantly impacts ocular health through various indirect mechanisms, including increased pollution, the spread of infectious diseases, extreme weather events and changes in allergens (Garg et al. 2024). These factors contribute to a range of ocular conditions, necessitating a comprehensive understanding of their implications. In addition, it facilitates the spread of vector-borne diseases, such as dengue and Zika, which can lead to ocular complications such as uveitis and retinitis (Garg et al. 2024; Waisberg et al. 2024). Furthermore, climate change increases the frequency of extreme weather events, leading to direct ocular injuries from trauma or chemical exposure during disasters (Wong et al. 2023). Variability in the climate contributes to a major influence on ocular health, making the relationship between climate change and eye health rather complicated (Auger et al. 2017; Garg et al. 2024, Waisberg et al. 2024).

Climate change has an intricate relationship with the ozone layer and ultraviolet (UV) radiation, with increased UV radiation as a result of ozone depletion resulting in notable effects on ocular and cutaneous health (Qassim et al. 2017). The important function of the ozone layer remains to protect the earth from dangerous UV radiation, and the eye is one organ susceptible to photochemical damage (Qassim et al. 2017). A study conducted among South African university students on their awareness of phototoxic effects as a result of ozone depletion revealed that 72.3% of the study sample of 1832 was aware of the effect of UV radiation (Oduntan et al. 2010). Furthermore, 47.1% of the study population had heard of cataracts before, and only 25.7% of students agreed that UV radiation exposure causes cataracts (Oduntan et al. 2010).

Research from the WHO indicates that 3.6 billion people now reside in regions that are extremely vulnerable to climate change. In addition, 2.2 billion individuals worldwide have either near- or far-sightedness, although not necessarily linked to climate change (Pachauri, Gomez-Echeverri & Riahi 2014; WHO 2019). At least 1 billion of these cases (nearly half) involved vision damage that was either preventable or that is still untreated (WHO 2019). These 1 billion people suffer from conditions such as cataracts, age-related macular degeneration and glaucoma, some of which may be influenced by environmental factors associated with climate change (Echevarría-Lucas et al. 2021; WHO 2019). Evidence suggests that the predicted prevalence of blindness and vision impairment in different communities will deteriorate because of the anticipated rise in eye diseases combined with the disruption of eye care services, affecting vulnerable populations inequitably (Echevarría-Lucas et al. 2021).

The understanding of climate change among health care professionals particularly concerning its health implications is of concern. Although it is acknowledged that climate change is a major global concern, not many are aware of how it affects health, particularly eye health. A scoping review on health science students’ preparedness for climate change reveals that more than 75.0% of the students in the included studies agreed that human activity is the main contributor to climate change, yet many also felt unprepared to deal with its impact on health (Ccami-Bernal et al. 2024). A Japanese study revealed a gap in awareness and action as only 32.6% of health professionals truly advise patients on climate change, despite 56.7% of them agreeing that it is important to do so (Igarashi et al. 2024). Studies reveal that medical students have an average awareness of how climate change affects overall health, but not of the risks to their eyes (George et al. 2024). There is a need for educational activities to improve understanding of climate-related health issues among healthcare providers in low- and middle-income countries (LMICs) (Ccami-Bernal et al. 2024; George et al. 2024; Igarashi et al. 2024).

The Lancet Countdown on Health and Climate report established in 2022 highlighted the fact that climate change affects health and urgent actions are required (Romanello et al. 2022). It will have several beneficial outcomes that include reducing the negative impact of health shocks and enhancing security and diplomatic risk (Romanello et al. 2022). Previous studies have highlighted the change in climate as a threat to health demanding acceleration in climate action that would result in a sequence of positive effects, including a reduction in the negative effects of health shocks and greater security and diplomatic autonomy (Igarashi et al. 2024). For over a decade, South Africa has developed a national climate change response policy, a national strategy for sustainable development and an action plan in response to climate-sensitive health risks (Van Der Bank & Karsten 2020). These actions contribute towards strengthening sustainable development goal (SDG) 13 on climate action and its action and SDG 3 to ensure healthy lives (United Nations 2023b).

Understanding the awareness and attitudes of future healthcare professionals towards these issues is critical for developing effective prevention and intervention strategies to mitigate their impact on population health. Healthcare professionals need to have the knowledge and skills to be able to identify as well as address climate change-related health issues, including those affecting the eyes (Dupraz & Burnand 2021). As climate change increasingly threatens global health, it is essential to assess awareness and understanding of its impacts across different sectors, including eye health, and facilitate collaboration for a holistic approach. Health science students represent the following generation of healthcare professionals who will play a crucial part in addressing climate change-related health issues. Understanding their knowledge and perceptions regarding the impact of climate change on eye health is vital for designing targeted educational interventions and preparing future healthcare providers to address emerging environmental health issues. The study aims to determine the knowledge and perception of final-year health science students on the impact of climate change on eye health.

## Research methods and design

This descriptive, cross-sectional, quantitative study was conducted at the University of the Free State (UFS). The study population involved all final-year students registered in the School of Health and Rehabilitation Sciences at UFS in 2024. The disciplines comprised by this School are Optometry, Physiotherapy, Occupational Therapy, Biokinetics and Dietetics. Participation was dependent on students’ willingness to be included in the study. A census approach was adopted whereby no sampling method was employed. All final-year undergraduate students enrolled in the School of Health and Rehabilitation Sciences at UFS in 2024 were invited to participate. This approach ensures comprehensive data collection from the entire target population, thereby enhancing the generalisability of the findings within the school. The study included all final-year undergraduate students registered in the School of Health and Rehabilitation Sciences at UFS in 2024 who provided informed consent while excluding researchers conducting the study and final-year students from other schools at UFS.

Data were gathered during June 2024 and July 2024 using a self-administered, semi-structured questionnaire containing closed-ended questions. The data collection tool consisted of three sections. Section A, Demographic Information; Section B, Perceptions of Climate Change and its Long-term Impact on Eye Health, contained four questions assessing participants’ beliefs about climate change, its anticipated impacts, the personal importance of the issue and their level of information on the topic; Section C, Awareness and Knowledge of Climate Change consisted of 18 statements addressing various possible influential factors of climate change on ocular health, including conditions such as cataracts, glaucoma, pterygium, allergic conjunctivitis and age-related macular degeneration. Participants indicated the degree to which they agreed with these statements using a 5-point Likert scale ranging from ‘strongly agree’ to ‘strongly disagree’.

The questionnaire used in this study was developed based on an extensive review of the literature and aligned with findings from previous studies focusing on knowledge and perceptions (Ofori et al. 2023). To ensure content validity, the instrument was reviewed and refined with the input of an expert in public health and a biostatistician, who assessed the relevance, clarity and appropriateness of the items for the study objectives. A pilot study was conducted with 12 participants from different departments within the school to evaluate the instrument’s feasibility, clarity and comprehensibility. Feedback from the pilot participants informed minor adjustments to the questionnaire, enhancing its validity and the results of the pilot were included in the main study.

Participant’s demographics, knowledge and perceptions were compiled using descriptive statistical methods. Categorical variables such as participant’s demographics, knowledge and perception items were summarised using frequencies and percentages, while medians and interquartile ranges (IQRs) were employed for numerical data to account for non-normal data distribution. The raw data, along with the auto-generated descriptive statistics, were extracted from Research Electronic Data Capture (REDCap) a secure, web-based application designed to support data collection, ensuring accurate and efficient data management. All the statistical analysis was conducted by a professional statistician, who utilised (SAS/STAT 15.2) software to ensure the robustness and validity of the results. No inferential statistics (e.g., chi-square tests or regression analyses) were conducted, as the study’s objective was descriptive. This comprehensive analysis approach allowed for a thorough examination of the data and provided insights into the knowledge and perceptions of the participants about the impact of climate change on ocular health.

### Ethical considerations

Ethical approval to conduct the study was obtained from the Health Science Research Ethics Council (HSREC) of UFS (reference no:UFS-HSD2023/2147/2603) and the Head of the School of Health and Rehabilitation Sciences provided a Gatekeeper’s approval letter. To comply with the *Protection of Personal Information Act* (POPIA), researchers obtained informed consent from the participants and gathered only essential personal information. An information sheet related to the study was provided to participants before they participated. The study adhered to the following ethical principles: Respect for individuals, privacy, justice and integrity, confidentiality, beneficence and non-maleficence. Participants were treated with respect, their privacy safeguarded and personal information kept confidential and used solely for research purposes. The study was conducted with honesty and transparency, which ensured equality in participant recruitment and handling of data, while it aimed to benefit participants and its impact on eye health and minimise any potential harm.

## Results

### Demographic characteristics

A total of 107 final-year health science students participated in the study from 124 students in the School of Health and Rehabilitation Sciences, resulting in a response rate of 85.30%. The age distribution revealed that most of the students (58.88%, *n* = 63) were between 18–22 years old, followed by those aged 23–27 years, 40.19% (*n* = 43), and a small fraction aged 28–32 years, 0.93% (*n* = 1). The gender distribution was predominantly female, with 82.24% (*n* = 88) identifying as such, while 17.76% (*n* = 19) were male. All participants were registered for qualifications within the School of Health and Rehabilitation Sciences and were in their final year of study. Specific qualifications included biokinetics, 14.95% (*n* = 16), dietetics, 7.48% (*n* = 8), occupational therapy, 27.10% (*n* = 29), optometry, 27.10% (*n* = 29) and physiotherapy, 23.36% (*n* = 25). Demographic characteristics of the participants are shown in Table 1.

**TABLE 1:** Demographic profile of participants.

Demographic characteristics	Category	Frequency	%
Age (years)	18–22	63	58.88
	23–27	43	40.19
	28–32	1	0.93
Gender	Female	88	82.24
	Male	19	17.76
Qualification	Biokinetics	16	14.95
	Dietetics	8	7.48
	Occupational therapy	29	27.10
	Physiotherapy	25	23.36
	Optometry	29	27.10

### Knowledge of climate change and its impact on eye health

The findings highlight varied perceptions among students regarding climate change’s specific effects on eye health. A majority (61.68%) agreed that increased global temperatures could influence cataract development, while 70.09% believed UV radiation is a controllable risk factor. In addition, 73.84% agreed that ozone layer depletion may contribute to cataract formation. Perceptions of air pollution’s link to glaucoma were mixed, with 35.52% agreeing, 29.91% neutral and 34.58% disagreeing. Similarly, 46.73% believed UV radiation could induce glaucoma, while 47.66% recognised malnutrition from climate change as a potential glaucoma risk. Most students (75.70%) agreed that outdoor workers are at higher risk of developing pterygium, and 71.03% felt reduced sun exposure could lower its incidence. A strong consensus (90.66%) associated seasonal changes with allergic conjunctivitis, while 74.77% agreed that excessive UV exposure could influence age-related macular degeneration onset. These results indicate significant awareness of climate-related ocular health risks although knowledge varied across specific conditions. Table 2 distinctly reports participants’ knowledge about climate change and its impact on eye health. Every participant answered the items, providing a comprehensive dataset.

**TABLE 2:** Knowledge among final-year health science students towards climate change and its long-term impact on eye health (*N* = 107).

Statements	Strongly agree		Agree		Neutral		Disagree		Strongly disagree	
	*n*	%	*n*	%	*n*	%	*n*	%	*n*	%
Increases in global temperature have an influence on the incidence and age of onset of cataracts.	16	14.95	50	46.73	32	29.91	8	7.48	1	0.93
UV radiation is a controllable risk factor for the development of cataracts.	27	25.23	48	44.86	23	21.50	8	7.48	1	0.93
Loss of the ozone layer may lead to the development of cataracts.	32	29.91	47	43.93	25	23.36	3	2.80	0	0.00
The development of glaucoma can be linked to air pollution.	8	7.48	30	28.04	32	29.91	33	30.84	4	3.74
Glaucoma can be induced by UV radiation specifically.	10	9.35	40	37.38	38	35.51	18	16.82	1	0.93
Malnutrition caused by climate change will contribute to the development of glaucoma.	13	12.15	33	30.84	33	30.84	24	22.43	4	3.74
People with outdoor occupations are more prone to develop pterygiums.	38	35.51	43	40.19	22	20.56	4	3.74	0	0.00
Less hours spent in the sun will reduce the onset of pterygiums.	26	24.30	50	46.73	24	22.43	6	5.61	1	0.93
An increase in allergic conjunctivitis each year is associated with seasonal changes.	52	48.60	45	42.06	9	8.41	1	0.93	0	0.00
Conjunctivitis can be the result of poor air quality.	39	36.45	49	45.79	11	10.28	8	7.48	0	0.00
Allergic conjunctivitis increases because of an increase in allergenic particles in warmer atmospheric conditions.	39	36.45	55	51.40	12	11.21	1	0.93	0	0.00
Excessive exposure to UV radiation can influence the onset of ARMD.	24	22.43	56	52.34	21	19.63	5	4.67	1	0.93
Sea levels are an environmental variable that can have an impact on the development of ARMD.	4	3.74	22	20.56	43	40.19	32	29.91	6	5.61
Air pollution related to traffic can influence the onset of ARMD	7	6.54	36	33.64	34	31.78	27	25.23	3	2.80

UV, ultraviolet; ARMD, age-related macular degeneration.

### Perception of climate change and its impact on eye health

The study revealed a high level of awareness among students regarding climate change, with 99.07% (*n* = 106) affirming their belief that climate change is currently taking place. Perceptions of its impact varied, with 42.06% (*n* = 45) anticipating effects soon, while 31.78% (*n* = 34) and 22.43% (*n* = 24) expected impacts within 10 and 5 years, respectively. Despite recognising its significance, 45.79% deemed it quite important and 19.63% very important, while 64.49% admitted feeling uninformed about climate change issues. Notably, 92.52% expressed concern if their ocular health was affected, with no students strongly disagreeing. Furthermore, 93.46% believed protecting their eyes outdoors could mitigate climate change risks on eye health, indicating an overall positive perception of its potential impact on ocular health and prevention strategies. Table 3 summarises the responses of participants regarding their perception of climate change and its impact on eye health. All participants responded to the items, ensuring a complete dataset for analysis.

**TABLE 3:** Perception among final-year health science students towards climate change and its long-term impact on eye health (*N* = 107).

Statements	Strongly agree		Agree		Neutral		Disagree		Strongly disagree	
	*n*	%	*n*	%	*n*	%	*n*	%	*n*	%
The chances of my eyes being affected by climate change in the next few years are great.	14	13.08	40	37.38	30	28.04	20	18.69	3	2.80
If climate change affects my eyes, I will be worried and concerned.	57	53.27	42	39.25	7	6.54	1	0.93	0	0.00
I believe protecting my eyes when I am outdoors will reduce the risk of my eyes being affected by climate change.	47	43.93	53	49.53	3	2.80	3	2.80	1	0.93
I have never heard or read anything encouraging me to protect my eyes from environmental factors.	17	15.89	22	20.56	10	9.35	31	28.97	27	25.23
Do you believe climate change is currently taking place on Earth?	Yes – 106	99.07	No – 1	0.93	-	-	-	-	-	-
Do you think you are well-informed about different issues related to climate change?	Yes - 38	35.51	No - 69	64.49	-	-	-	-	-	-
When do you believe climate change will impact the Earth?	Soon – 45	42.06	In the next 5 years – 24	22.43	In the next 10 years – 34	31.78	Never – 4	3.74	-	-
How important is the issue of climate change to you personally?	Very important – 21	19.63	Quite important – 49	45.79	Not very important – 31	28.97	Not important at all – 6	5.61	-	-

## Discussion

These findings highlighted the significant awareness and varied perceptions among final-year health science students regarding the effect of climate change on eye health. The results also highlighted a critical need for enhanced educational initiatives to better inform students about the specific environmental factors influencing ocular health. The findings from the study revealed a notably high degree of awareness among final-year health science students regarding climate change and its possible effects on eye health. This awareness aligns with global trends indicating increased recognition of climate change among healthcare practitioners and students (La Torre et al. 2023).

Nearly all participants affirmed the occurrence of climate change, demonstrating widespread acknowledgement, consistent with previous studies (Nigatu, Asamoah & Kloos 2014). This high level of awareness is consistent with students in Ethiopia, where 77.5% of participants were aware of climate change and its impacts as well (Nigatu et al. 2014). Participants who were involved in the study collectively believed in climate change, indicating a high degree of awareness that is consistent with global trends in climate change recognition. Regarding the perceived timeline for the impact of climate change, students’ individual concerns were inconsistent with research findings from Bangladesh, where university students often overlooked the psychological effects of climate change despite being aware of its physical impacts. This indicates a gap in comprehensive understanding (Rahman et al. 2018).

The self-reported level of students being informed about climate change was relatively low, with more than 50% of the students demonstrating that they did not feel well informed. The discrepancy in self-reported knowledge underscores the necessity for improved climate change teaching resources. Research such as that conducted by Maxwell and Blashki (2016) highlighted how critical it is to incorporate teaching on climate change into health curricula. More than half of the participants agreed that they were aware of the potential impact of climate change on ocular health occurring within the next few years, which aligns with surveys conducted by the Open Society Foundation despite the survey conducted among the general population (Narawad 2024). The perceptions and opinions obtained from the survey conducted by The Open Society Foundation in 30 countries highlighted how much more aware the general population is becoming about these risks (Narawad 2024). However, the few participants who disagreed or strongly disagreed with the notion could be because of awareness regarding the link between climate change and ocular health, and this indicates a need for increased public education on the phenomenon.

Most of the participants in this study agreed that they would be worried and concerned if their eyes were affected by climate change, while no percentage of students strongly disagreed. A significant number of students also agreed that they believed protecting their eyes outdoors could decrease the risks of climate change effects on eye health consistent with other studies (Kansal & Khan 2023). According to the United States Environmental Protection Agency, ozone layer depletion-related UV radiation and other climate-related factors can affect eye health (Rahman et al. 2018). Furthermore, WHO also emphasises how UV affects eye health causing eye conditions such as cataracts (WHO 2003). Students’ positive perceptions were reflected in their concern for eye health and belief in preventative efforts against the repercussions of climate change on eye health. To better educate students on the details of climate change and its effects on health, there appears to be a need for more focused educational activities, as shown by the knowledge gap.

Participants firmly believed that wearing protective gear when outside could lower their chances of acquiring climate change-related eye health problems. This belief was in line with current research that underscored how crucial eye protection is in reducing the effects of environmental dangers made worse by climate change (Maxwell & Blashki 2016). According to research done in Taiwan by Chen et al. (2021), the public believes that sunglasses that block out UV radiation are effective in reducing the risk of cataracts and other solar UV radiation-related ocular damages, and their findings are similar to our study.

Participants further believed that the occurrence and onset of cataracts were influenced by increased global temperatures. This perception may be explained by the documented association between global warming and increased UV radiation exposure, which is known to be a major environmental risk factor in the formation of cataracts (Yadav 2019). Excessive UV radiation exposure is a significant risk factor for cataract development, and this risk is predicted to increase when global temperatures rise because of an increase in UV radiation, especially in areas that are closer to the equator (Borges-Rodríguez, Morales-Cueto & Rivillas-Acevedo 2023). Mahendra and Andari (2022) found that, especially in warmer regions, exposure to intense UV radiation sped up the opacity of lenses. Furthermore, a study involving geographically disparate populations of India revealed that increased outdoor exposure was associated with an increased risk of cataract development (Vashist et al. 2020). This confirms the research findings, especially for those who strongly agreed or concurred. When asked if an increase in global temperatures could affect the incidence and age at which cataracts develop, a significant portion of participants remained neutral. This may indicate that they did not fully understand the obvious connection between cataract development and rising global temperatures. Age, heredity, lifestyle and certain medical diseases such as diabetes are all significant risk factors for cataract development (Atif et al. 2018). Some may have found it difficult to link the onset of cataracts to climate change because of this intricacy without more precise information.

Previous research shows that more efforts are required to enhance knowledge and promote the use of protective sunglasses, besides using other methods of protection from UV radiation, such as hats and umbrellas (Alebrahim et al. 2022). Despite these precautionary measures, it is possible that not all participants in our study were aware of UV exposure as a variable risk factor, which accounted for the inconsistent findings. The high percentage of participants who concurred may be a result of various movements and efforts encouraging sun protection and raising public awareness of the dangers of UV radiation. Concerning the low proportion of respondents who strongly disagreed might point to the necessity of more effective public health initiatives that highlight the unmistakable connection between UV exposure and the development of cataracts (Alebrahim et al. 2022).

A significant number of participants agreed that there was a link between ozone layer depletion and cataract development. This was consistent with well-established scientific data, such as a cross-sectional study by Vashist et al. (2020) that examined the relationship between UV radiation exposure and cataract development. They found that an increased amount of UV radiation reaching the earth’s surface was a considerable risk factor for cataract development. Mixed responses in perception had been found in our study on the link between glaucoma and air pollution. While other research had not established any conclusive evidence, contrasting studies such as Chua et al. (2019) suggested a possible link between prolonged exposure to air pollution and an increased risk of glaucoma. Students’ knowledge may have varied because of inconclusive data despite several researchers suggesting an association between glaucoma and air pollution.

Various responses were given regarding whether UV radiation may cause glaucoma. In contrast to cataracts, the formation of glaucoma has not been conclusively related to UV radiation; however, there was research evidence that proposed UV exposure may increase oxidative stress in ocular tissues, which may exacerbate circumstances that could influence the evolution of glaucoma (Wang et al. 2014). As there was insufficient evidence to establish a causal relationship between UV radiation and glaucoma, educational programmes should instead focus on eye conditions with well-established associations to UV exposure such as cataracts, AMD, pterygium and dry eye disease while excluding glaucoma as a primary focus. Similarly, the link between malnutrition and glaucoma remains inconclusive and should be approached with caution in public health messaging.

The results showed a mixed belief regarding the link between the development of glaucoma and malnutrition caused by climate change. There was an extensive amount of research on the consequences of climate change on food security and the impact of malnutrition on overall health, but there was little solid proof connecting these two factors to glaucoma in particular (Misra 2014). As Sommer (2008) pointed out, there was a well-established connection between malnutrition, specifically vitamin A deficiency, and ocular health problems such as night blindness and dry eye. Direct evidence linking glaucoma and malnutrition was unclear. Rather than being directly caused by dietary deficits, glaucoma is a complex disease principally connected with hereditary variables and intraocular pressure (Sommer 2008).

Our results suggested that there was an extremely strong agreement that individuals who worked outside were more likely to acquire pterygiums. Pterygium development has been closely linked to UV light exposure, which is typical in outdoor occupations (Zhou et al. 2016). Ding, Zhang and He (2024) addressed the pathogenesis of pterygium and mentioned UV exposure as a major risk factor. The strong agreement among participants regarding the link between outdoor occupations and pterygium development reflects their perceived knowledge, which is consistent with existing evidence highlighting UV radiation as a key risk factor. This understanding aligns with findings by Li et al. (2018), who demonstrated the biological impact of UV exposure on ocular structures in individuals with pterygium. Most of the participants believed that spending less time outdoors would lower the risk of developing pterygiums. Numerous studies have shown that UV radiation is one of the most influential environmental determinants for developing pterygium (Rezvan et al. 2018). A study conducted by Vashist et al. (2020) also found that pterygium was considerably more common among those who had higher levels of sun exposure, particularly in those who lived in coastal locations. The results were in line with previous studies on the connection between UV radiation and pterygium development, showing that a decrease in the amount of time spent in the sun correlated with a lower incidence of pterygium onset (Moran & Hollows 1984).

Most participants strongly agreed that an increase in allergic conjunctivitis each year was associated with seasonal changes; this was consistent with studies such as Sarwar et al. (2022) that discussed a correlation between seasonal changes and increased allergic conjunctivitis cases, supported by lower vitamin D levels and higher Immunoglobulins E (IgE) levels in patients with seasonal allergic conjunctivitis. A substantial amount of knowledge existed among participants regarding the seasonal nature of allergic reactions affecting the eyes. The participants also believed that the development of conjunctivitis was significantly influenced by poor indoor and outdoor air quality, whereas no participants disagreed with this statement. The conjunctiva could become inflamed as a result of exposure to pollutants, allergens and irritants, which could cause both acute and chronic eye disorders (Chang et al. 2012).

A study conducted by Chang et al. (2012) investigated the relationship between air pollution and patients treated without staying overnight in hospital for non-specific conjunctivitis. It was found that exposure to high levels of air pollution considerably raised the risk of contracting conjunctivitis, especially in children and those with underlying ocular disorders (Chang et al. 2012). A notable number of participants agreed that an increase in allergic conjunctivitis was associated with an increase in allergenic particles. This was following studies such as Ziska et al. (2011) that found that increased temperatures and high levels of carbon dioxide enhanced pollen production, which in turn led to the exacerbation of allergic conditions such as allergic conjunctivitis.

There was a broad agreement regarding the influence of UV radiation on the onset of age-related macular degeneration (ARMD). The high degree of agreement demonstrated that students were aware of UV exposure as an ARMD risk factor, which was in line with results from meta-analyses and systematic reviews (Patil & Gupta 2022). Regarding sea levels that had an impact on the development of ARMD, a mixed response was obtained from participants. Rising sea levels brought on by climate change were linked to modifications in UV radiation and air quality, both of which have an effect on ARMD and the variability in responses from participants highlighted how complicated and indirect the connection is between ARMD and the rise in sea levels (Wong et al. 2023).

Chang et al. (2019) looked at the relationship between ARMD and air pollution. The study discovered a link between the exposure to fine particulate matter such as nitrogen dioxide, which is a common form of traffic-related pollution and the risk of acquiring ARMD (Chang et al. 2019). This study further supported the theory that prolonged exposure to air pollution adversely affects the ocular health of ageing populations (Chang et al. 2019). While a substantial number of participants acknowledged this relationship, a significant number of participants were neutral or disagreed. This may reflect the variability in their awareness or the complexity of understanding how environmental conditions could impact ocular health. The results obtained accentuated the disparities in final-year health science students’ knowledge and comprehension of the relationship between environmental factors specifically climate change and eye health.

The University of the Free State’s health sciences departments’ curricula were not reviewed for this study, making it impossible to evaluate in totality the course material concerning environmental impact. Comparing this study to similar studies proved to be more challenging because many of the studies did not address eye health or studies were conducted in a broader population and not among students. This may also restrict this study’s findings to be generalised for national and international comparative analysis.

## Conclusion

The degree of awareness among final-year health sciences students regarding how climate change affects ocular health varied. Key contributors such as exposure to UV radiation, air pollution, extreme weather, seasonal changes in allergens and food and water insecurity were demonstrated by the participants in their awareness. These factors highlight the various ways in which climate change may affect eye health. This highlighted the necessity of improving educational initiatives to close the knowledge gaps. Inter-professional education on climate change and its health implications could enable future healthcare professionals to advocate for and implement preventive measures effectively. Implementing comprehensive strategies is essential to lessen the detrimental effects of climate change on ocular health by integrating targeted health promotion and climate-health education into existing curricula across health sciences disciplines. In addition, raising awareness about the importance of UV protection and nutritional interventions could contribute to reducing the burden of climate-related ocular diseases. This study further underscored the urgency of addressing climate change within the realm of public health and optometry education. By equipping students with thorough knowledge and fostering informed perceptions, we can better prepare them to tackle the multifaceted challenges posed by climate change to eye health. It is proposed that comparable studies be carried out in other disciplines with much emphasis on climate change and overall health to mitigate future risks. The results of this study on climate change and eye health provide baseline data for future studies.
